# The Military Training of the Officers of the R.A.M.C. (T.F.)

**Published:** 1910-04-16

**Authors:** 


### The Royal Army Medical Corps Section.

### THE MILITARY TRAINING OF THE OFFICERS OF THE R.A.M.C. (T.F.).

It is quite excusable to consider a thorough knowledge of medical science as the only necessary qualification for Army medical officers, seeing that the functions of the medical corps are, primarily, the prevention of disease, and, secondarily, the care of the sick and wounded; but a little consideration of the full scope of their duties shows that there is need for a much wider range of knowledge than at first sight appears. This is made manifest when one considers the various details of work that fall to their share. They have the professional supervision of all sanitary measures and precautions, the collection of the sick and wounded, their removal from the front by suitable transport, the maintenance and discipline of these sick and wounded while under their charge, and the compilation of their records, both general and professional. Further they have very important executive duties in connection with their own R.A.M.C. units, such as the provision of food, clothing, and necessaries for their men, the care and management of the transport allotted to them, the arrangements for camping, and the bringing of their units into the space allotted to them on the line of march, so that it is no exaggeration to say that R.A.M.C. officers have to carry out almost the same duties as officers of other units with the single exception that they do no fighting.

The above remarks have been prompted by the recent issue to the field units of the Territorial Medical Service of a small pamphlet entitled "Royal Army Medical Corps Training. Part III. Military Training," the object of which is to make clear to medical officers that this is a very important branch of knowledge that they must make themselves acquainted with outside the actual hospital and professional duties they may have to perform, seeing that non-acquaintance with it detracts from their efficiency and affects the interests of the Army they are attached to. Although this pamphlet has come to the R.A.M.C. (T.F.) more as a guide than an instruction, it is advisable to draw attention to it and briefly summarise what it lays down.

In insisting on the need of military training for Army medical officers the pamphlet points out that professional duties form only a limited part of their work, and that even these cannot be carried out properly "without a certain general knowledge of military science, especially as regards the administration of an army in the field," for on every side they are brought into touch with the general work of the Army. In the same way it is imperative that they be acquainted with the tactical principles underlying the employment of the different fighting units of a force, otherwise they cannot grasp the orders transmitted to them, nor can they properly provide them with the necessary medical aid for meeting the casualties of the battlefield. As to the means to be followed in carrying out this individual training of officers in military subjects, stress is laid on the following:—

(1) Staff tours, (2) war games, (3) regimental exercises and indoor schemes, and (4) essays. Upon all these subjects useful observations and suggestions are made. In our opinion there can be no question that for the proper study of medical problems in the field staff tours specially designed for the instruction of medical officers afford the best educational facilities. While carrying them out medical officers are brought face to face with definite tactical situations, and have to meet them by the actual disposition or movement of the medical units at their disposal, so that they gain valuable experience and learn promptness of action. We agree, too, with the opinion that the presence of medical officers on staff tours that are designed merely for the study of strategical and tactical problems is also advantageous to the military directing staff, for they can point out that certain of the procedures decided on may in their execution be accompanied by such evident risks to health, and would throw such an undue strain on the medical services that they should not be undertaken, thus indicating the value of medical knowledge in the conduct of military operations, and emphasising the need for a medical member of the headquarters staff in time of war. What has been said of staff tours applies equally to war games, which are conducted on the same principles, and afford excellent scope for a detailed study of medical problems in the field, and for the disposition and movement of medical units to meet distinct tactical situations.

The lectures on the principles of staff duties in the field suggested in the pamphlet show what an extensive range of subjects the military training of medical officers covers, but they all bring out very important details of knowledge that are most essential for the efficient working of the medical service attached to an army. It is, for instance, very necessary that medical officers should recognise the independence of the various departments of the Army and their relation to the fighting troops, and should understand the broad general principles of the chain of command, so that they may know with whom they have to deal on the various matters affecting their duties, and how they can obtain what they require for their units as regards *personnel*, material, horses, and any other requirements. Again, the pamphlet points out that the Army Medical Service is part of an organisation which is maintained solely for the special purpose of fighting. In view of this the course of lectures includes such a general outline of strategical and tactical operations as will enable a medical officer to formulate the most suitable arrangements in connection with such operations, and as this subject carries with it an explanation of the characteristics and methods of work of the various arms and of the different marching and fighting formations, including simple time and space calculations, as well as the essential points to be remembered in arranging movements, it furnishes the information necessary for the fitting of units into their proper place on the line of march or elsewhere Another very useful branch of knowledge dealt with in the course of lectures is the working of the Army's lines of communication, and the general principles to be followed in selecting the routes for the evacuation of the sick and wounded, and the provision of stationary and clearing hospitals. Lastly, there is included instruction in the writing of field messages and reports, and in map reading and simple field sketching, so that medical officers may be able to understand any orders they may receive, to frame properly such orders as they may have to issue, to describe the proposed site of a hospital by reference to a map, and to send in a rough sketch of any proposed building or hospital.

The above may seem a formidable list of subjects to be mastered, but we cannot too clearly recognise that modern warfare is dependent for its success on a high standard of organisation and skill on the part of those engaging in it, a statement that applies with equal force to the Army Medical Service. Of its duties and responsibilities we cannot take too wide a view, for its efficiency means often a great deal to the Army that it serves. This should be an incentive to its medical officers to perfect themselves in a knowledge of Army organisation, of field work, and the best methods of utilising the general medical resources in an area of operations, remembering that a training which confines itself merely to first-aid and ambulance drill does not embody the whole duty of a medical service, the standard of efficiency aimed at being the ability also to bring large bodies of sick and wounded rapidly, and with an absence of interruption and confusion, to the general hospitals at the base.

